# ‘Feminization’ of physician workforce in Bangladesh, underlying factors and implications for health system: Insights from a mixed-methods study

**DOI:** 10.1371/journal.pone.0210820

**Published:** 2019-01-11

**Authors:** Puspita Hossain, Rajat Das Gupta, Phyoe YarZar, Mohamed Salieu Jalloh, Nishat Tasnim, Ayesha Afrin, Nahitun Naher, Md. Tarek Hossain, Taufique Joarder, Syed Masud Ahmed

**Affiliations:** 1 BRAC James P Grant School of Public Health, BRAC University, Dhaka, Bangladesh; 2 University Research Co., LLC, Yangon, Myanmar; 3 eHealth Africa, Freetown, Sierra Leone; 4 WAVE Foundation, Dhaka, Bangladesh; 5 Infectious Diseases Division, International Centre for Diarrhoeal Disease Research Bangladesh (icddr,b), Dhaka, Bangladesh; 6 Health Systems and Population Studies Division, International Centre for Diarrhoeal Disease Research Bangladesh (icddr,b), Dhaka, Bangladesh; 7 FHI 360, Dhaka, Bangladesh; Aga Khan University, KENYA

## Abstract

**Background:**

Bangladesh is currently faced with an emerging scenario of increased number of female physicians in the health workforce which has health system implications. For a health system to attract and retain female physicians, information is needed regarding their motivation to choose medical profession, real-life challenges encountered in home and workplaces, propensity to choose a few particular specialties, and factors leading to drop-out from the system. This exploratory mixed-methods study attempted to fill-in this knowledge gap and help the policy makers in designing a gender-sensitive health system.

**Methods:**

Three-hundred and fifteen final year female medical students from four purposively selected medical colleges of Dhaka city (two each from public and private colleges) were included in a quantitative survey using self-administered questionnaire. Besides, 31 in-depth interviews with female students, their parents, and in-service trainee physicians, and two focus group discussions with female students were conducted. Gender disaggregated data of physicians and admitted students were also collected. Data were analysed using Stata version 13 and thematic analysis method, as appropriate.

**Results:**

During 2006–2015, the female physicians outnumbered their male peers (52% vs. 48%), which is also supported by student admission data during 2011–2016 from the sampled medical colleges, (67% in private compared to 52% in public). Majority of the female medical graduates specialized in Obstetrics and Gynaecology (96%). Social status (66%), respect for medical profession (91%), image of a ‘noble profession’ (91%), and prospects of helping common people (94%) were common motivating factors for them. Gender disparity in work, career and work environment especially in rural areas, and problems of work-home balance, were a few of the challenges mentioned which forced some of them to drop-out. Also, this scenario conditioned them to crowd into a few selected specialties, thereby constraining health system from delivering needed services.

**Conclusions:**

Increasing number of female physicians in health workforce, outnumbering their male peers, is a fact of life for health system of Bangladesh. It’s high time that policy makers pay attention to this and take appropriate remedial measures so that women can pursue their career in an enabling environment and serve the needs and priorities of the health system.

## Background

In recent times in medical profession, females have started outnumbering their male colleagues, in both developed and developing countries–a phenomenon sometimes referred to as ‘the feminization of medicine’ [1–3].Worldwide, 32% of the medical graduates are females [4]. For instance, in the USA the number of female physicians increased from 27% in 1983 to 48% in 2011 and in Canada, there has been a fourfold increase within a span of 40 years [5,6]. Similar trends have also been observed in Netherlands and most of its European neighbours, extending as far as Malta and Israel [5,7,8]. The South-east Asian countries such as Thailand, China, Malaysia, and Indonesia are also catching up, where female physicians now comprise around 50% of the total number of registered physicians [9–12].

Factors such as humanistic appeal of medicine, socioeconomic status and liberal family environment, early inspiration from a family role model (doctor), parental expectations, and financial prospects have been identified as motivations to enroll in medical schools [13,14]. In case of females, additional motivations include social prestige emanating from the profession and a high value in the marriage market [15], cultural preference for female doctors in conservative community [16], and intrinsic factors like willingness to help poor people arising from an altruistic attitude [17]. Female physicians have preference for few selected specialties (for example, Obstetrics and Gynaecology and Paediatrics as opposed to Surgery) [4], mainly due to competing life priorities (for instance, marriage, child bearing, and family obligations in a patriarchal society), and work-life imbalance. [7,18]. They also face challenges relocating to rural job postings because of the existing attitude towards women working in an unknown environment dominated by men [19]. However, their narrow choice of specialty and limited ability to relocate to rural areas has health system implications such as poor availability of primary health care (PHC) services.

Consistent with this global trend, Bangladesh is also undergoing a rapid change in sex composition of the physicians. As recent as in 2013, 31% of all registered physicians were females in the country and their number is increasing day by day [20]. Similar trend is observed among the population of medical students as well, for example, 48% of the medical students admitted into the 2015–16 session were female [21]. This phenomenon is throwing a big challenge before the female physicians for advancing their career, including choice of specialties which are often disconnected to the needs and priorities of the health services and medical education [22].

Furthermore, the numbers of female health workers (especially physicians and nurses) working in the rural areas are becoming fewer [23], which can further exacerbate in coming days due to ongoing influx of women in medical and allied professions. Another problem is attrition or drop-out from the profession with consequent effects on already starved (of qualified professionals) health services. For example, high attrition rate among female physicians have been reported in Bangladesh who never enter into the profession after graduation [24]. This is also commonly seen in Pakistan where many female physicians eventually end up becoming housewives [25].

The secular increase in the number of female physicians is a relatively new phenomenon in Bangladesh and there is dearth of comprehensive and gender disaggregated data on this topic. This information is needed for planning the production of physician workforce in future according to the needs and priorities of health services, and allocate to different specialties as delineated in the country’s human resources for health (HRH) strategy. This study aimed to fill-in these knowledge gaps e.g., regarding the level and trend of sex composition of physicians during 2006–2015, factors that motivate female students to study medicine including the reasons for their parents’ preference for this, choice of particular specialties, challenges faced by them both as students as well as professionals in work and family lives. The findings are expected to inform necessary changes for a gender-sensitive and enabling work and living environment that facilitate female physicians’ retention and career progress, including balanced production of specialists based on the needs and priorities of the health systems [4].

## Methods

### Conceptual framework

**Fig 1:**
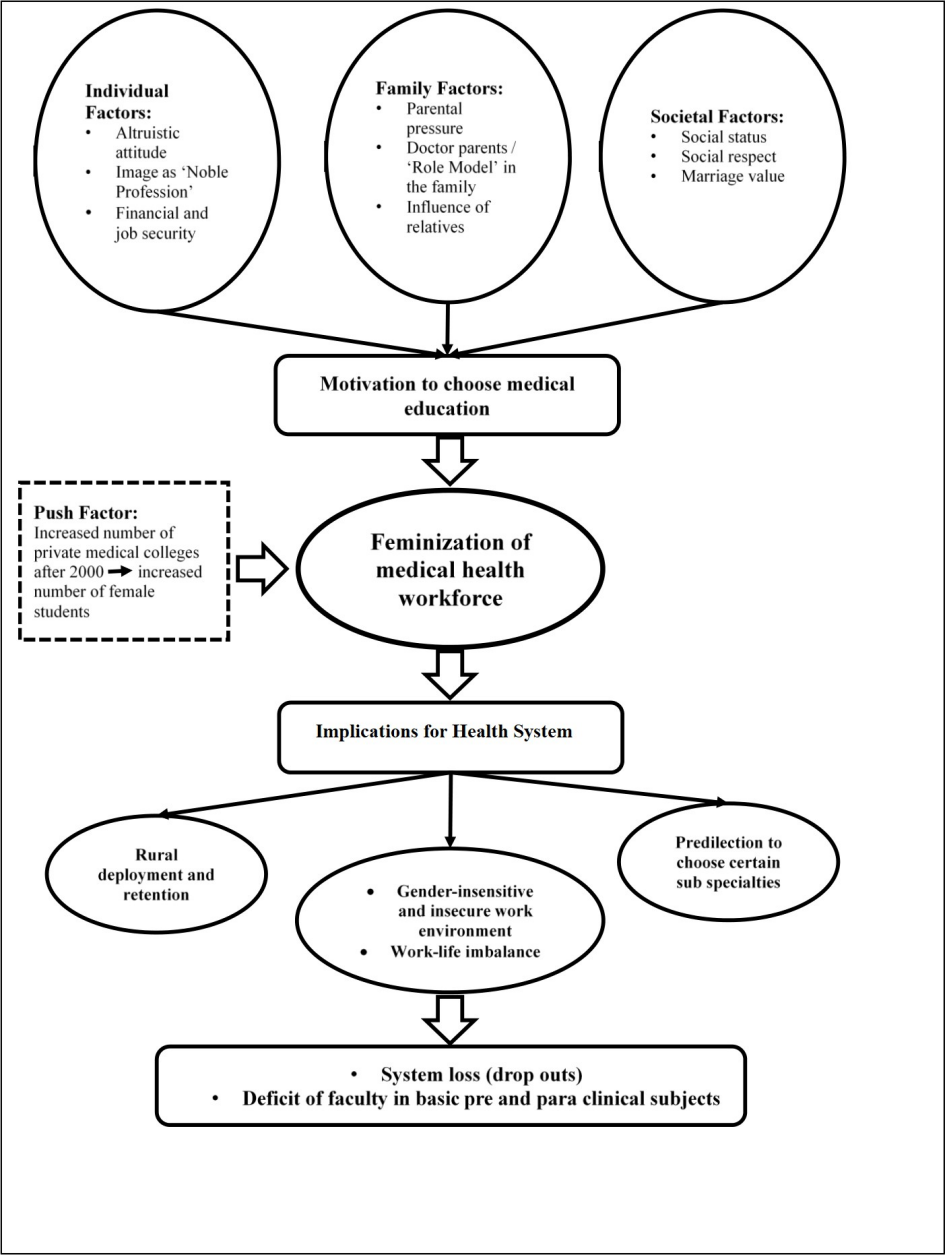
Conceptual framework.

The conceptual framework guiding this study is based on current knowledge on the topic (Fig 1). It shows that there are different factors that motivate females to embark on studying medicine. These include personal factors like fulfilling a much cherished dream to help people by being part of a noble profession, gaining social status and respect, and achieving financial security. Familial factors like influence of the parents (more so when either or both are physicians) and relatives are especially important, as they act as family ‘role models’. Besides, better socio-economic status [13,14] and marriage market prospects (of getting a ‘better’ groom) also attract women and their parents to pursue the study of medicine [15]. However, as discussed above, there are quite some challenges that the female medical students and physicians face in their academic and professional life in a male-dominated society, interfering with balance of their work-home lives. This phenomenon has some implications for the health system of the country as well e.g., problems with deployment and retention in the rural areas due to security and other reasons, substantial attrition resulting from the latter, and crowding in to certain preferred specialties which provide a kind of ‘comfort zone’ (especially for the reproductive age women and women with children) for them. This occurs at the cost of scarcity in other needed specialties in pre-, para-, and non-clinical subjects (Fig 1).

### Study design

This was a cross sectional exploratory study using a triangulation mixed-methods approach. Data were collected from primary and secondary sources, during 17 November—3 December, 2016, by students of the study team (n = 6). Both methods were used in order to obtain precise information on the research topic as well as to triangulate the findings. Secondary data were collected to find out the level and trend of feminization of physician workforce in Bangladesh. Quantitative survey using a structured questionnaire was done to elicit factors motivating female students to enroll in medical schools and their specialty preferences along with the implications on health systems. Besides, qualitative methods such as focus group discussion and in-depth interviews were done to explore challenges faced by the female students and physicians, and parent’s motivation in preferring medicine as a field of study for their daughters (Table 1).

**Table 1:** Summary of the methods, respondents, objectives addressed and some variables/sample questions.

Methods	Respondents	Objectives addressed	Variables/Sample questions asked
***Quantitative***			
(self-administered questionnaire)	Final year female medical students (n = 315)	To find out factors motivating the female medical students to enroll in medical colleges and choosing particular specialties; health system implications	Socio-demographic and economic characteristics; factors motivating enrollment; factors influencing career choices and specialty preferences; drop-outs and shortage of faculty in pre-, para-, and non-clinical subjects
***Qualitative***			
a) Focus Group Discussion (FGD)	2 Sessions of FGD done with 11 female medical students	To find out details of challenges faced by the female students	
b) In-depth Interview (IDI)	Final year female medical students (n = 16)	To find out details of challenges faced by the female students	*Do you find any difference between faculty treatment of male and female students; any difference between male and female student attitudes*, *behavior*, *performance; expect any difference between treatment of males and females in work places*, *differences in future career plan…*
	Parents of female medical students (n = 5)	To find out the parent’s opinion about preferring medicine as a field of study for their daughters	*Feelings of being parents of a female physician; factors influencing choice of study for daughters; influence of parent(s) being a physician on study choice of daughter(s);perception about their future career choice/specialty choice…*
c) In-depth Interview (IDI)	In-service trainee physicians (n = 10)	To explore the real-life experiences of the physicians in service	*What was expectations before admission; how these changed*, *if any*, *after you became a physician; what do you think about reasons for drop out; your experience in current work as trainee*

### Sample and data collection

This study was done as part of the MPH course requirement and sample for FGDs and IDIs were selected conveniently, using personal connections and networks of the MPH students in their respective medical colleges. Thus, primary data were collected from final year female medical students of four selected medical colleges in Dhaka city (two public and two private) (n = 315), a sample of female in-service trainee physicians from these medical colleges (n = 10), and a sample of parents of students participating in the survey (n = 5) (Table 1).

### Primary data collection

Both quantitative (classroom survey) and qualitative data were collected from final year female students in selected medical colleges. The classroom survey used a pre-tested, self-administered questionnaire. First, permission was obtained from the principal of the sampled medical college few days before the actual survey was conducted. The date and time of the survey was unknown to the students and the faculty, except the principal. This was done to minimise the preconception among participants which might have arisen due to discussion of the survey topic among themselves. On the day of survey, the respective authority was approached with a request to allow the study team to run the survey before the classes began at a particular time. Only the female students were requested to stay in the classroom. The objectives and process of the study was briefly introduced by the study team, followed by instructions on how to fill-in the self-administered questionnaire. They were then requested to participate in the survey on a voluntary basis, assuring them of no consequences in case of non-participation. The study team approached a total of 325 students in the four medical colleges out of which 315 students consented to participate (response rate 97%). The questionnaires were then distributed to those consenting, and the survey took around 30 minutes to complete.

The qualitative data were collected using pre-tested guidelines. Two FGDs with female students were conducted, in each of the sampled public and private medical colleges. For in-depth interviews, a sub-sample of the participating students, and a sample of in-service trainee physicians from the sampled medical colleges, were selected and interviewed discreetly at their place of preferences (mostly the dormitory). Besides, five parents of the participating students (conveniently selected) were also interviewed in-depth. The survey questionnaire and qualitative guidelines are submitted as supporting files (S1 File and S2 File).

### Secondary data collection

Secondary data were collected to find out level and trends of sex distribution of physicians over a 10 year (2006–2015) period and admitted students over a six year (2011–2016) period. Bangladesh Medical and Dental Council (BMDC) provided data on registered physicians and the student data was provided by the respective offices of the medical colleges. Sex specific data was searched in the BMDC website (http://bmdc.org.bd/) and manually counted for each year based on the first and last registry number of a year. In case of missing profiles of physicians, verification was done with BMDC and counted again in each related year. The Bangladesh College of Physicians and Surgeons (https://www.bcpsbd.org/) provided data on post graduate memberships (MCPS) and fellowships (FCPS) in seven specific specialties. These were Medicine, Surgery, Paediatrics, Obstetrics and Gynaecology, Ophthalmology, Psychiatry and Anaesthesia.

### Data analysis

Quantitative data were entered into MS excel, cleaned and then analyzed using Stata version 13.0. For the primary data, summary statistics of the age of the respondents were described using mean, and other statistics related to general socio-demographic characteristics were described as proportion. For categorization of the specialties, Anaesthesia, ENT (Ear Nose Throat), Ophthalmology, Orthopaedics, Radiology, and Surgery were grouped into ‘Surgery’. Internal medicine and Psychiatry were grouped together as ‘Medicine’. Finally, Anatomy, Biochemistry, Physiology, Pathology, and Microbiology and Forensic medicine were grouped together as ‘Basic science’. The other specialties such as Clinical research, Public Health, Paediatrics, and Obstetrics and Gynaecology remained as standalone categories. For showing comparison between public and private medical students, Pearson’s χ^2^ test or Fisher’s Exact test was used for categorical variables as appropriate and two sample *t*-test was used for continuous variables. A *p*-value of less than 0.05 was considered to be statistically significant.

For the qualitative data, transcription and translation was done within 24 hours of data collection. The transcripts were read at least three times to get familiar with it. Different a priori codes were prepared while preparing the semi-structured IDI and FGD guidelines. Some codes emerged from repeated reading of the transcripts and inductive codes were prepared. The a priori codes were also divided into sub-codes (Table 2). Then the data was clustered, compared and categorized, and data display was prepared to identify pattern. The data were analysed by the student investigators [PH, RDG, PYZ, MSJ, NT and AA], under the close supervision of the mentors [NN and MTH] and the supervisors [SMA and TJ]. Data validity check was done by triangulation, member feedback and looking for researchers’ effect. Triangulation was ensured by looking into data from IDIs and FGDs, as well as by selecting respondents from both public and private medical colleges. The qualitative data was checked by all the members of the research team [PH, RDG, PYZ, MSJ, NT and AA, NN, MTH, TJ and SMA]. To reduce researcher’s effect on the setting, time was given to build rapport with the respondent and intentions were clarified during data collection. To minimize the effect of setting on researcher, the field notes and memos were checked by all the members of the research team. Better quality data was given more importance in drawing conclusion.

**Table 2:** Codes and sub-codes used for qualitative data.

Codes	Sub-codes	Type of code
Motivating factors for parents	• Helping the people	• A-priori
	• Dignity/ Prestige	• A-priori
	• Financial reason	• A-priori
	• Work independently	• Inductive
	• Role model in the family	• A-priori
	• Having a physician in the family	• Inductive
Challenges for women working in the medical sector	• Challenges faced by medical students and intern doctors	• A-priori
	• Coping up with the obstacles	• A-priori
	• Perception of dropout among students and doctors	• Inductive
	• Perception about effect of drop out	• A-priori
	• Methods of reducing drop out of female from health workforce	• A-priori
Reasons for preferred place to work	• Access to social and family networks	• A-priori
	• Adventure & recreational opportunities	• Inductive
	• Access to Information and Communication Technology	• Inductive
	• Road Infrastructure	• Inductive
	• Community belonging	• Inductive
	• Good working condition, including better infrastructure and equipment and good hygienic condition	• A-priori
	• Workplace security	• A-priori
	• Opportunities for children	• Inductive
	• Opportunities for own continuing education	• A-priori
	• Opportunities for partner/spouse	• Inductive
	• Rural lifestyle	• A-priori
Reasons for preferred sub specialties	• Gender distribution and Opportunities	• A-priori
	• Working Environment	• A-priori
	• Exposure to Technical Skills	• Inductive
	• Female role in Bridging Specialty Gap	• Inductive
	• Social and Cultural reasons	• A-priori

### Ethical considerations

The study protocol including the data collection tools were approved by the Ethical Review Committee of the BRAC James P Grant School of Public Health, BRAC University. No invasive procedure was involved in this study., Informed written consent was taken from the respondents before data collection began (using self-administered questionnaire for quantitative data, and guidelines and checklists for qualitative data). Also, the respondents were given full freedom to skip any questions or withdraw themselves from the study at any given point without providing any explanation. The quantitative data was given unique identification numbers before entering into the software to maintain anonymity. The qualitative data was also analysed anonymously.

## Results

The results are presented according to the two methods of data collection used:

### A) Findings from the quantitative survey

#### Level and trend of sexual composition of physicians (2006–2015) and admitted students (2011–2016)

**Fig 2:**
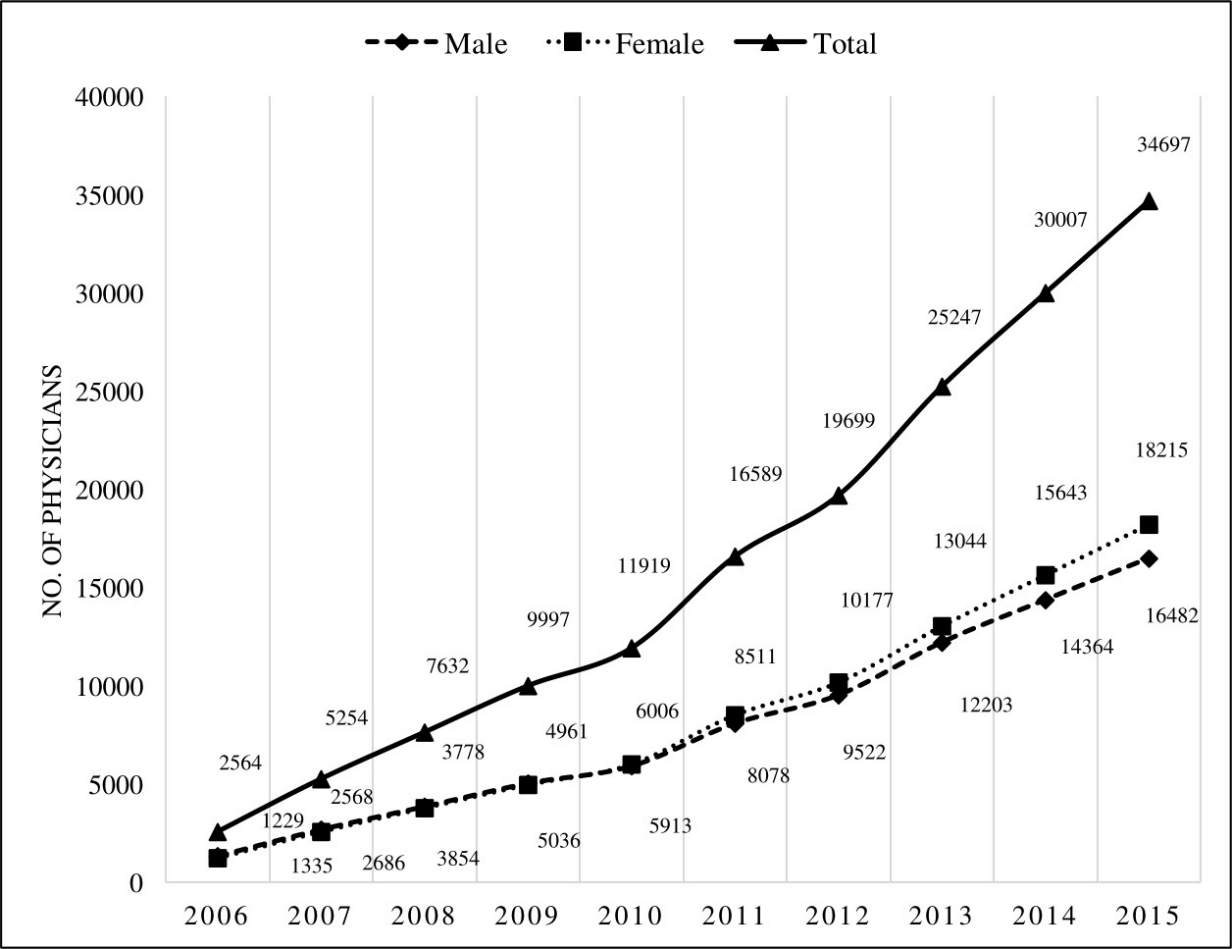
Gender distribution of physician (2006–2015), cumulative number.

**Fig 3:**
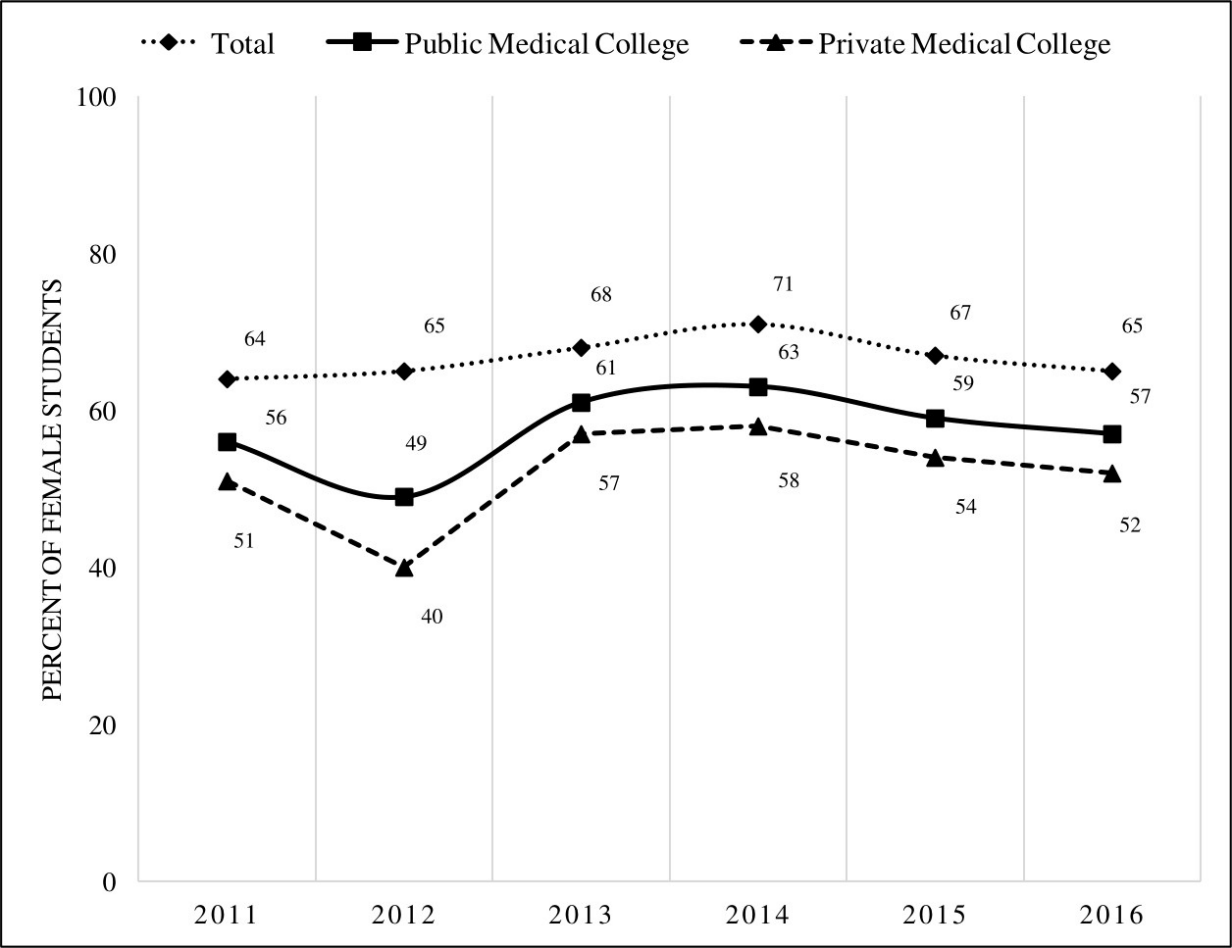
Distribution of female students by selected medical colleges (2011–2016).

Out of a total of 34, 697 registered physicians (cumulative) during the ten-year period of 2006–2015, 48% (16,482) were male and 52% (18,215) were female (Fig 2). From 2011 to 2016, majority of the students being admitted in the two public and private medical colleges were female (range: 40–58% and 64–71% respectively) (Fig 3).

**Fig 4:**
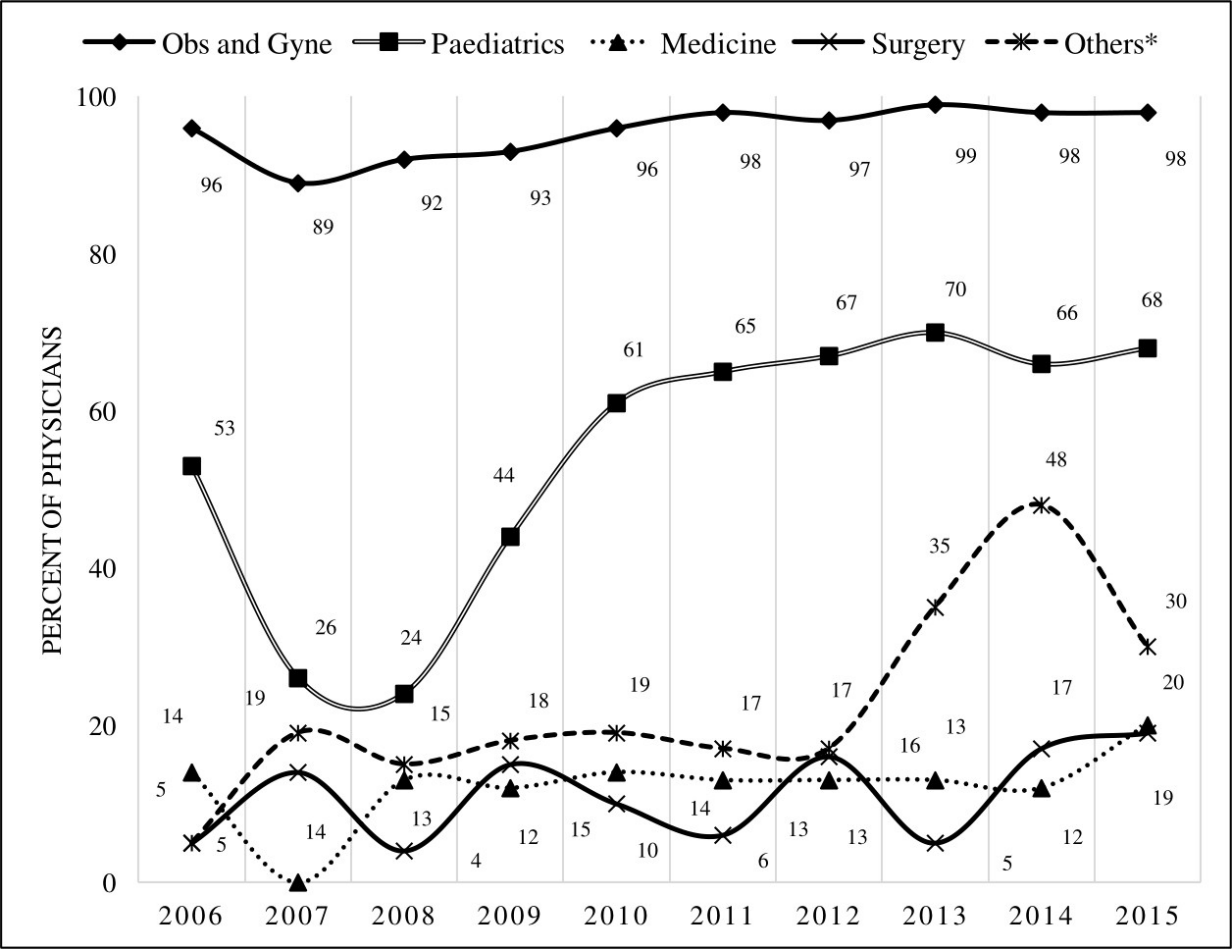
Distribution of female physicians according to specialization (2006–2015).

Medicine and Surgery were the most popular specialties amongst males in the ten-year period (87%); for females this was Obstetrics and Gynaecology (96%), followed by Paediatrics (57%). In other specialties, such as Ophthalmology, Psychiatry and Anaesthesia, the proportions of female were found to be 28%, 24% and 11%, respectively (Fig 4).

#### Characteristics of the final year female medical students

In case of primary survey, 207 (66%) of the respondents were from public and 107 (34%) were from private medical colleges (Table 3). Majority of the students (93%) were from urban background, more so in case of students from the private medical colleges (*p* = 0.01). Students of private medical colleges came from relatively educated families- their fathers and mothers had post-graduate education in greater proportion than those of the public medical colleges (61% vs. 48% and 44% vs. 26%, respectively), and also, from relatively affluent families (monthly family income >1,00,000 BDT, *p*<0.001).

**Table 3:** Socio economic characteristics of the respondents (N = 315).

Variable	Female medical students			
	Public	Private	Total	*Chi-square* *p-*value
	N = 207 n (%)	N = 108 n (%)	N = 315 n (%)	
**Age in years**	22.71±0.80	22.46±0.86		0.01 (*t-test)*
**Permanent residence**				
Urban	187 (90.3)	106 (98.1)	293 (93.0)	0.01
Rural	20 (9.7)	2 (1.9)	22 (7.0)	
**Highest level of education of father**				
≤8 years of schooling	5 (2.4)	2 (1.9)	7 (2.2)	0.11
9–12 years of schooling	38 (18.4)	11 (10.2)	49 (15.6)	
Graduates	64 (30.9)	29 (26.9)	93 (29.5)	
Postgraduates	100 (48.3)	66 (61.0)	166 (52.7)	
**Highest level of education of Mother**				
≤8 years of schooling	11 (5.3)	3 (2.8)	14 (4.4)	<0.001
9–12 years of schooling	89 (43)	28 (25.9)	117 (37.1)	
Graduates	53 (25.6)	30 (27.8)	83 (26.4)	
Postgraduates	54 (26.1)	47 (43.5)	101 (32.1)	
**Profession of father/ male guardian**				
Medical doctor	17 (8.2)	15 (13.9)	32 (10.2)	0.27
Business	42 (20.3)	29 (26.9)	71 (22.5)	
Service	138 (66.7)	59 (54.6)	197 (62.5)	
Others	10 (4.8)	5 (1.6)	15 (4.8)	
**Profession of mother/ female guardian**				
Medical doctor	7 (3.4)	9 (8.3)	16 (5.1)	0.05
Business	7 (3.4)	7 (6.5)	14 (4.4)	
Service	58 (28)	19 (17.6)	77 (24.4)	
Housewife	129 (62.3)	67 (62)	196 (62.2)	
Others	6 (3)	6 (5.6)	12 (3.9)	
**Monthly income of the family (in BDT)**				
<40,000	63 (30.4)	9 (8.3)	72 (22.9)	<0.01
40,000–100,000	132 (63.8)	69 (63.9)	201 (63.9)	
>100,000	12 (5.8)	30 (27.8)	42 (13.2)	

#### Reported reasons for choosing to study medicine

The most important individual factors motivating the females to choose medical education were social respect accorded to the profession (94%), perceived opportunity to help people (94%), serving a ‘noble’ profession (91%) and fulfilling a ‘childhood dream’ (73%). Significant difference was present between the public and private medical respondents across two motivational factors: social respect to the profession (*p* = 0.02) and scientific nature of study (*p* = 0.03), the percentage was more so for the private medical students than the public medical students (Table 4).

**Table 4:** Female medical students’ reported reasons for choosing medicine as a field of study (N = 315).

	Type of Medical College			
	Public	Private	Total	*two-sample test for proportion* *p*-value
	N = 207 n (%)	N = 108 n (%)	N = 315 n (%)	
**Individual Factors**				
Opportunity to help people	195 (94.2)	100(92.6)	295(93.7)	0.57
Respect earned	199 (96.1)	97(89.8)	296(94.0)	**0.02**
Noble profession	192 (92.8)	94(87.0)	286(90.8)	0.09
Childhood dream	151 (72.0)	80 (74.7)	231 (73.3)	0.83
Study science	118 (57.0)	75(69.4)	193(61.2)	**0.03**
Job security	135 (65.3)	70(64.8)	205(65.2)	0.89
Financial security	140 (67.6)	70(64.8)	210(66.6)	0.61
**Familial Factors**				
Parental pressure	89 (43.0)	43 (39.8)	132 (41.9)	0.58
Parents are doctors	19 (9.18)	18 (16.7)	37 (11.8)	0.05
Influence of friends/relatives/role model	80 (38.7)	35 (32.4)	115 (36.5)	0.27
Illness of friends/ relatives	41 (19.8)	18 (16.7)	59 (18.7)	0.50
**Societal Factors**				
Social status	143 (69.1)	66(61.1)	209 (66.4)	0.15
Social competition	92 (44.4)	37 (34.2)	129 (41.0)	0.08
Contribution to society	174 (84.1)	92 (85.2)	266 (84.4)	0.79
Marriage value	36 (17.4)	18 (16.7)	54 (17.1)	0.87

Among the familial factors, parental pressure (42%) and influence of friends/ relatives and role models (37%) were dominant. Although not significant, more respondents from the private medical colleges (17%) were motivated to choose medicine because of their physician parents (Table 4). Among the societal factors, contribution to society and social status of the profession were mostly reported (84% and 66% respectively). In total, only 17% reported its marriage value and there was no significant difference between the two types of colleges (Table 4).

#### Reported preferences for specialization

Obstetrics and Gynaecology and ‘Surgery’ were the most common preferred subspecialties (27%), followed by Medicine (20%) and Paediatrics (15%). The predilection towards ‘surgery’ was more among the private medical college students (35% vs. 22%, *p* = 0.01), while Obstetrics and Gynaecology (32% vs. 19%, *p* = 0.01) and Paediatrics (18% vs. 9%, *p* = 0.04) were preferred by those from the public medical colleges (Table 5). Very few female students (<5%) preferred to study Basic Sciences, Public Health and Clinical Research.

**Table 5:** Female medical students’ reported preference of specialties (N = 315).

	Female medical students and intern doctors			
Preferred specialty	Public	Private	Total	*two-sample test for proportion* *p-*value
	N = 207 n (%)	N = 108 n (%)	N = 315 n (%)	
Surgery	46 (22.2)	39 (36.1)	85 (27.0)	**0.01**
Obstetrics and Gynaecology	66 (31.9)	20 (18.5)	86 (27.3)	**0.01**
Medicine	42 (20.3)	21 (19.4)	63 (20.0)	0.86
Paediatrics	37 (17.9)	10 (9.3)	47 (14.9)	**0.04**
Basic Science	6 (2.9)	5 (4.6)	11 (3.5)	0.52
Clinical Research	2 (1.0)	5 (4.6)	7 (2.2)	0.05
Public Health	1 (0.5)	5 (4.6)	6 (1.9)	**0.02**
Not Yet Decided	7 (3.4)	3 (2.8)	10 (3.2)	0.53

### B) Findings from the FGDs and IDIs

These are described under relevant themes:

#### Parent’s motivational factors for sending their daughters to medical colleges

In-depth interviews reveal that the most important motivation underlying parents’ preference for their daughters to study medicine was the charitable nature and social prestige of the profession. According to parents, a girl can help the people, contribute to society and stand in a higher social position through this profession. They thought that it is also safer and more suitable profession for girls.

“*First of all*, *it’s a noble profession*. *Secondly*, *there are plenty of opportunities to help and contribute to the lives of people when their lives are in danger*. *This is also the most respected and secured profession for girls in our society*. *That’s why I preferred this profession for my daughter*.*”*

---mother of a private medical college student

According to the parents, the diverse work opportunities in medical profession offer an important advantage for girls to work independently in their field of choices, giving attention to her family if necessary.

*“In medical profession*, *a girl can work independently; it has a flexibility which is very necessary for a girl because she has to raise her children; she has other obligations for her family…”*

---mother of a public medical college student

Having a doctor in the family (‘role model’) was considered another important factor by the parents. They perceived that a doctor in the family can help them in taking appropriate decisions in case of medical emergencies. Besides, doctors are considered meritorious and hold a higher status in the society; therefore, becoming the parent of a doctor was a matter of pride to them.

*“My daughter will be a doctor and serve the people and she will be established. So when I say it to my family members or others then they say that I am a successful parent*. *When I hear this I feel very proud.”*

---father of a public medical college student

Sometimes the wishes of the parents take the form of indirect mental pressure to compel the students to study medicine. One in-service trainee physician remarked:

*“Actually I deeply dislike that parents force their children*. *Children can have their own dream; after all it’s their own life… parents should support their children for what they want to be… I suffered much in my student life!”*

---Female in-service trainee physician

#### Challenges for women working in the medical sector

We further explored the challenges medical students face during their academic and training period. These are presented below.

**i) Socio-cultural norms**

The female medical students perceived that the most important challenges come from the society and their families. Firstly, they mentioned that after marriage the situation seems to change drastically for the female students and physicians in terms of family support. For example, their husbands and in-laws’ expect that they would prioritise their families over their careers and their workplaces. One respondent explained this situation by saying,

*“As most girls cannot maintain both career and family life equally*, *they have to compromise* …*the situation in Bangladesh is such that*, *you have to give more preference to married life over career*.”

---medical student, public medical college

An in-service trainee expressed this more vividly:

*“I had to compromise with myself (*regarding administrative and social issues*) because that’s what you have to do*. *How it happened I don’t know* … *I got habituated*. *In the beginning I felt bad*, *I still feel bad*, *but now I think that there is no point in expressing these things*.*”*

The social construct of the ‘right’ age of marriage is also another issue that creates challenges for the female students and physicians alike. As one of the respondents said,

*“We must get married within 30 years of age to settle down whereas it does not matter if a man marries at the age of 35years*.”

---medical student, public medical college

The third major issue that came up was the shift duties especially in the night. According to them, most of the families do not want them to work night shifts.

*“…That aunty was proudly saying that she didn’t let her daughter work at night*, *rather she could stay at home not doing any job*.

---medical student, public medical college

This is echoed by an in-service trainee physician as well:

*“Often it is seen that when a girl is doing night duty- society asks a lot of questions*, *even when there is family support…I myself am afraid of what if I don’t get similar support and can’t go ahead with my career as I have planned* …”

**ii) Gender disparities**

Almost all the female students said that in spite of being equally qualified, society including some of the female patients consider female physicians as less competent compared to male physicians. According to the female students, their male peers used to think them as 'less productive' in their professional life, though they had done better in their student life. However, parents opined that in the past, society used to discriminate between male and female physicians; but this attitude is changing now a days.

The gender disparity is also prevalent in case of career choices, job posting, duty shifts, promotion, and their status as physician. The respondents have mentioned that the society, even their teachers, believed that boys are more talented and competent than girls. One respondent highlighted this,

*“Everyone always thinks that the boys are brainier than us*. *Even our teachers think in this way*. *If in a family there’s a daughter and a son*, *it’s obvious- they want the girl to be doctor and the boy to be engineer*.*”*

---medical student, private medical college

Another respondent said that this attitude of looking at them as inferior to male physicians even extended to the patients:

*“…They say that public can address us as ‘sister’ as we are female*, *females are like sisters*. *But they do not understand that public address a professional as ‘sister’* … *They do not understand that people address us ‘sister’ taking us as a nurse*. *They post lots of statuses (in Facebook) and try to make fun of us”*.

---medical student, public medical college

The career of husband gets preference over the wife’s, even if they are both physicians. For example, as reflected in the statement below

*“A male who has already established his career in the country*, *will not be willing to leave everything behind to accompany her wife if she plans*, *for example*, *to study abroad; while under the same circumstance*, *it’s the girl who has to go with husband without any question… …*.*”*

---Female in-service trainee physician

One of the administrative issues that came up in the discussion was the issue of promotion:

*“In terms of promotion*, *authority always tries not to promote girls*. *They think girls won’t be able to handle the job responsibilities*, *as they have family to take care of*.*”*

---medical student, public medical college

**iii) Workplace insecurity**

Most of the respondents preferred to work in the health centers located in the urban and peri-urban areas, even if they preferred public sector jobs. The main reasons given were having access to social and family network, better opportunity for professional development including post graduate training, workplace security and also being habituated to the urban lifestyle during the long years in the medical colleges and hospitals during study. They viewed lack of autonomy and patriarchal attitude of the society as a challenge to work in the rural areas. They opined, if conditions improve, more females would be interested to work in the rural areas.

*“In most of the villages, up until now, females are not given autonomy… the right to do things on their own … so, when a female doctor goes there to work, they think she will do whatever they command*. *This mentality of the rural people must be changed.”*

---medical student, public medical college

According to the respondents, the workplace security in the rural health facilities is less than the urban health facilities, which hinders female doctors’ interest in seeking posting in the rural area.

*“There should be security at the workplace…if there is any mistake while providing care, people literally would tear apart the doctor*. *They tease and harass the doctors, specially the female doctors. If you can provide security, the female doctors will be motivated to work in the rural areas.”*

---medical student, private medical college

Students mentioned that they are not encouraged by families to take public sector jobs and subsequent rural postings because of this security issue; often the resistance is unsurmountable for them.

#### Perceived implications by students (and physicians)

The students perceived that, given the above challenges, they will have to face certain realities in their professional life. These are presented below.

**i) Difficulty in taking up rural postings**

The main difficulty the students perceived was related to the condition of the residential facilities. From anecdotal evidence from their seniors in rural jobs, they perceived the rural facilities to be inadequate (congested, dirty, insecure) to live, and therefore, not congenial for taking up rural postings. The administrative issues were mostly reported by the public medical college students, but the social security issue was reported by both public and private medical college students. One respondent mentioned:

*“Working in the urban area will be easier for me. After entering into marital life*, *it will be easier for me to maintain the family.”*

---medical student, public medical college

**ii) Propensity to choose few clinical specialties**

The students speculated that due to societal norms and gender issues, and difficulty with balancing work-home life, they may have to choose only a few areas for specialization (e.g., Obstetrics and Gynaecology and Paediatrics) like their predecessors, and most of them are unwilling to become teachers of basic medical subjects. However, they thought that in future things may change with changes in society’s attitude. To quote:

*“People in our country still think that every female doctor will be a gynaecologist* … *I think upcoming female doctors will pursue different specialties and overcome some stigmas…”*

---medical student, public medical college

**iii) Perceived effects on the health system**

Most of the students observed that compared to the number of female students, there are not as many practicing female physicians. This is creating a gap, especially in the conservative rural areas where female doctors are needed more for managing the female patients. One respondent said,

*“Actually in our country female doctor is needed*, *and most females prefer female doctors*. *Now those who are becoming MBBS doctors but not pursuing career*, *our patients are not getting their services*, *they are being deprived*.*”*

---medical student, public medical college

They also mentioned that when a physician drops out, it is an economic loss to the country given the investment required to produce a doctor. One of the in-service trainee physician mentioned:

*“Now we see that females are being admitting more in the medical colleges, but at the end of the day, they are dropping out more. It is a waste*. *A lot of good doctors are not going into career… dropping out will create a gap in the health sector.”*

An in-service trainee physician explained the scenario in detail:

*“I think females drop out more*. *Females have many kinds of bindings*. *Most females get married during their internship*. *So once they get into the family life*, *a lot of things they have to compromise*, *and eventually the study is hampered*. *Sometimes it is seen that the subject or career that she wants to choose is not possible because of family*, *then she opt for more traditional subjects such as Obstetrics and Gynaecology*. *Some families don’t want the females to pursue career*. *That’s why I think females flourish more during undergraduate level*, *but do not do that well in post-graduation*.*”*

Another in-service trainee physician presented the issue of drop-out differently:

*“I think*, *first of all*, *females need to change their own mentalities*. *Females have to be strong willed to pursue their own career and establish in life and manage both family and social aspects as well* …*so that females can pursue their dreams and not drop out”*.

## Discussion

This study was done to explore the emerging phenomenon of ‘feminization’ of the physician workforce in Bangladesh including its underlying reasons and the challenges it pose to their professional and family lives. Besides, the health system implications of this phenomenon such as problems with rural deployment and retention, system loss (attrition), and shortage of faculty in certain pre- and para-clinical medical subjects were also investigated. These issues are discussed thematically against the backdrop of the evidence presented including its implications for HRH planning in future.

### Implications of privatization of medical education after 2000: Money mattered more than merit

Beginning 2000, medical education began to be privatized officially in Bangladesh and recognized by the Bangladesh Medical and Dental Council (BMDC), the regulatory body for approval and licensing medical colleges. Currently there are 69 approved private medical colleges in the country (out of total 105 in the public and private sector) offering 63% of total available seats. The private medical colleges charge exorbitant tuition and other fees, almost 100 times higher than the public medical colleges. Interestingly in the early years, these colleges did not have to follow stringent admission criteria like those for the public medical colleges. Thus, any student with science and affluent family background got admitted. This is also reflected in the findings which show that the students from the private medical colleges were mostly coming from affluent families compared to public medical colleges, plausibly so because of the high tuition and other fees charged by the former [26]. Again, data in the study reveal that high probability of climbing up the social ladder, high social value and prestige of the profession, better prospects in the marriage market, and opportunity to fulfill ‘dreams of the parents’ by money if not by merit were some of the key underlying reasons. This is consistent with what have been observed in other countries as well [27,28].

It may be mentioned here that the admission criteria has changed over the last ten years. Now, all aspiring students of science background irrespective of gender or other characteristics, and securing a combined minimum score from the secondary and higher secondary school examinations (total 12 years of education) have to undergo a uniform, centralized admission test. There is a minimum cut off score (for quality control) to be eligible for admission, and the students are ranked according to merit based on scores in the admission test. Again, the medical colleges are also ranked, the public over the private, starting from the best one in the public to the last one in the private sector. The students get selected based on their score and preferences. Thus, there is no barrier for females to get admitted in the public or private medical colleges as it depends on their merit ranking. However, only students who are at the top of merit ranking (usually the first 3,000) can get admitted to the public medical colleges. After all the allocated seats in the public medical colleges are filled up, the rest of the students become eligible to get admitted in the private medical colleges based upon merit ranking. But the catch is: only those who are financially solvent to pay the high admission fees in these colleges can get admitted. So, there is the possibility (and it practically happens) that students with lower merit ranking in the test but from wealthier family have a greater chance of being admitted. These students may be females in greater number, for reasons mentioned above.

### Motivational factors for female medical students: Financial security and job security are not the whole story

Serving a noble profession was mentioned by the overwhelming majority of female respondents, overriding issues such as financial or job security. This may be due to the fact that in patriarchal societies like Bangladesh, financial responsibility for raising and maintaining a family is viewed as the sole purview of men [29]. So, it is assumed that women are less concerned about financial return from the profession unlike their male peers. This was reiterated by the parents’ expressed preference to the perceived altruistic nature of the profession. In the developing countries, altruism has been proposed as a way to improve the performance of the nursing students [30]; the same can be experimented in case of medical profession as well. Similar findings were also noted in the OECD regions where helping people was the most important factor for motivating the female medical students to take up the profession [31].

### Health system implications of ‘feminization’ of medical workforce: Problems of ‘system loss’, rural deployment, and shortage of faculty in pre- and para-clinical subjects

In a conservative society like Bangladesh, the need of female physicians cannot be over emphasized. Interestingly enough, the greater proportion of female medical students do not correspond with the proportion of female physicians who are currently active in the profession, as mentioned in the interviews. In the process, many ‘drop out’ and become ‘inactive’, reducing the pool of available physicians on which the health system can draw. We need more detailed data on this ‘system loss’ over time to gauge the exact magnitude of the problem and plan for future medical workforce.

The gender equality and equity aspect play a big role in the drop out from medical workforce. Gender equality refers to women having equal opportunity and access to resources as men and gender equity about being fair in terms of professional opportunity irrespective of men and women [32]. The societal belief about the appropriate role for men and women dictates fairness and justice in the professional working structure which sheds light on the disproportion that exists between the number of women in medical education and the number of women in power [32,33].

Studies show that even though more and more women are coming into medical profession, the socio-cultural norms related to gender roles and expectations of a male-dominated society assume that they are primarily responsible for maintaining home and family life. As such, balancing work and personal life as well as career advancement is a great challenge for women in the medical profession, if necessary support does not come from the family [22,34]. Besides, the long time required to build an expertise and career in medical profession, while maintaining family, becomes untenable for the female physicians who often fail to reach the leadership position [34]. Combination of these factors sometimes compels a substantial proportion of female physicians to’ drop out’ of the system and become ‘inactive’. This ‘system loss’ from health sector is not uncommon and has also been observed in other countries, for example Pakistan [19] and Japan [35].

Regrettably, the gender norms and attitudes of patriarchal society like that of Bangladesh are not always conducive for a woman to take up rural postings. Personal security concerns in the workplace especially in the rural areas, lack of women-friendly work environment, and problems with work-life balance are some of the underlying reasons which discourage them to take up rural postings and remain in the system. The personal security at workplace is an overwhelming issue for all female health care providers (doctors, nurses), especially for those working in the public sector [36,37]. This situation makes it challenging for the female physicians to remain in rural postings for long [23,38], which is also observed in India [39]. The situation may further exacerbate in future as more and more women will graduate from the medical schools in the coming days.

Another concern is the propensity of female physicians to develop career in a few specific clinical specialties, thus contributing to the current situation of shortage of required faculty e.g., in the pre- and para-clinical subjects. Currently, the country fills only 25% of the allocated posts of teachers required for basic medical subjects [40]. Similar situation is also observed in India where females are entering the medical schools in greater numbers, but their representation in different academic sub-specialties are not proportional [41].

### Limitations of the study

First and foremost, this paper originated from a student project conducted for fulfilling partial requirement of an MPH course, and thus constrained with respect to time and resources. Thus, the findings may not be generalisable for the rest of the female medical students, in-service trainees and physicians in Bangladesh. However, trend analysis of the physician data over time confirmed the emerging ‘female face’ of the physician workforce in the country. Using mixed methods data, this exploratory study presents a fair picture of the scenario and provides insights on motivation of females for enrolling in medical education, challenges of the profession in a not-so-friendly work environment, and subsequent impact on the health systems. For a comprehensive picture of ‘feminization’, further studies are needed using a nationally representative sample and time series data, and more qualitative studies to document their coping and surviving strategies in the system over time, and how these impact on health systems.

## Conclusion

The emerging phenomenon of ‘feminization’ of physician workforce has thrown a new challenge before the health system of Bangladesh. The policy makers and practitioners should be cognizant of this, and plan and implement appropriate remedial measures for creating an enabling environment to attract and retain them in the system. The flip side of the coin is the fact that this phenomenon may affect the health system of Bangladesh in a positive direction by increasing its responsiveness because female physicians are found to take better care of patients compared to their male colleagues [42]. Also, pro-active measures are needed for increasing the pool of female specialists in the pre- and para-clinical subjects to cater to increasing needs of the newly established medical, nursing and paramedic institutions in the country.

## Supporting information

S1 File Survey questionnaire. (DOCX)

S2 File Qualitative guidelines. (DOCX)

S3 File Quantitative dataset. (DTA)
