# Supplementary material for: ‘Feminization’ of physician workforce in Bangladesh, underlying factors and implications for health system: Insights from a mixed-methods study
Source: PLoS One. 2019 Jan 11;14(1):e0210820. doi: 10.1371/journal.pone.0210820 (PMC6329528; doi:10.1371/journal.pone.0210820)
Supplement: S1 File — (DOCX) [file pone.0210820.s001.docx]

| **Questionnaire on ‘Factors affecting job and career preferences of final year female Medical students and intern doctors in selected medical college hospitals in Dhaka City’**  Serial No: *(to be filled by researcher) __________* Date: *(dd/mm/yy)*___________ |
| --- |

**Give Tick (√) mark to the box at the right side of the option you prefer
Section A: Background Information**

Name_____________ Name of the medical college_____________

A1. Age in years_____________

A2. Type of medical college: 1. Public ☐ 2. Private ☐

A3. Year of Study: 1. New 5^th^ Year ☐ 2. Final Professional Examinee ☐ 3. Intern Doctor ☐

A4. Marital status: 1. Single ☐ 2. Married ☐ 3. Divorced ☐ 4. Widowed ☐

A5. Religious affiliation: 1. Muslim ☐ 2. Hindu ☐ 3. Christian ☐ 4. Buddhist ☐ 5. Other ☐

A6. When you were 1-15 years, where did you spend most of your life?:
1. Rural ☐ 2. Small Town ☐ 3. District Town ☐ 4. Divisional City ☐

A7. Where is your high school located, from where you passed SSC? :
1. Rural ☐ 2. Small Town ☐ 3. District Town ☐ 4. Divisional City ☐

A8. Where is your college located, from where you passed HSC? :
1. Rural ☐ 2. Small Town ☐ 3. District Town ☐ 4. Divisional City ☐

A9. Profession of Mother/ Female guardian?:
1. Doctor ☐ 2. Business ☐ 3. Service ☐ 4. Housewife ☐ 5. Others ☐

A10. Profession of Father/Male guardian?:
1. Doctor ☐ 2. Business ☐ 3. Service ☐ 4. Unemployed ☐ 5. Others ☐

A11. Where your parents live now? : 1. Village ☐ 2. Small Town ☐ 3. City ☐ 4. N/A ☐

A12. What is your monthly family income (in Taka)?: __________________________________

A13. Number of family member you are living with: __________________________________

A14. What are the highest education level of your father and mother?

| **A14.a. Highest education level of your Father** | **A14.b. Highest education level of your Mother** |
| --- | --- |
| ☐ 1. Never attended school | ☐ 1. Never attended school |
| ☐2. Primary school (Class1 -8) | ☐2. Primary school (Class1 -8) |
| ☐3. SSC | ☐3. SSC |
| ☐4. Diploma/Vocational level | ☐4. Diploma/Vocational level |
| ☐5. HSC | ☐5. HSC |
| ☐6. Bachelor level | ☐6. Bachelor level |
| ☐7. Postgraduate level | ☐7. Postgraduate level |
| ☐8. Others; specify……….. | ☐8. Others; specify……….. |

**Section B: Choosing medical studies**

What are some of the key factors that influence you in choosing medical school? Can you select/ tick **(√)** YES if any of the factors highlighted influence your decision and NO if it is not applicable. Please mention the reason beside why it is Yes / No:

| No | Factors influencing career choice | Yes | No |
| --- | --- | --- | --- |
| **B1.1 Individual factors** | |  |  |
| 1 | Opportunity to help people |  |  |
| 2 | Honor/prestige |  |  |
| 3 | Noble profession |  |  |
| 4 | Childhood dream |  |  |
| 5 | Personal prosperity |  |  |
| 6 | Scientific study |  |  |
| 7 | Intellectual challenge |  |  |
| 8 | Variety of carrier |  |  |
| 9 | Job security |  |  |
| 10 | Financial security |  |  |

| **B1.2 Familial factors** | | Yes | No |
| --- | --- | --- | --- |
|  | Parental pressure |  |  |
|  | Doctor parents |  |  |
|  | Influence of friends/relatives /role model |  |  |
|  | Illness of friends/ relatives |  |  |

| **B1.3 Societal factors** | | Yes | No |
| --- | --- | --- | --- |
|  | Social status |  |  |
|  | Social competition |  |  |
|  | Working with people |  |  |
|  | Contribution in society |  |  |
|  | Marriage value |  |  |
|  | No specific reason |  |  |

**Section C: Preferred Sub--Specialty Choice**

**C1.** What are your top two (2) preferred choices for specialization?

Please mark**(√)**against your 1^st^ and 2^nd^ preferred choices as 1 and 2 from the list given below, Please choose only one subject first choice and only one subject as second choice:

| **Specialty choice** | **1^st^ Choice** | **2^nd^ Choice** |
| --- | --- | --- |
| 1. Anaesthesia |  |  |
| 1. Basic Science (Anatomy, Biochemistry, Physiology, Pathology, Microbiology, Pharmacology) |  |  |
| 1. Clinical Research |  |  |
| 1. ENT (Ear Nose Throat) |  |  |
| 1. Forensic Medicine |  |  |
| 1. Internal medicine (includes sub-specialties) |  |  |
| 1. Obstetrics and Gynaecology |  |  |
| 1. Ophthalmology |  |  |
| 1. Orthopaedics |  |  |
| 1. Paediatrics (includes sub-specialties) |  |  |
| 1. Public health |  |  |
| 1. Radiology |  |  |
| 1. Surgery (includes all sub-specialties) |  |  |
| 1. Psychiatry |  |  |
| 1. If Not decided **(Skip section C2, go to section D)** |  | |

**Section C2: Motivations for Sub-Specialty Choice**

**C2.** What motivated you to choose a preferred specialty?

**In this section, if you have answered to Question C1 above please mark (√) against two options, for each of the categories given; please note categories:**

**C 2.1 Organization Factors**: Related to mode specialty training and future expectations

**C 2.2 Socio-cultural Factors**: Related to community, religious values and cultural beliefs

**C 2.3 Intrinsic Factor**: Related to your personal interest and beliefs

**C 2.1** For **organization related factors** listed below; please mark **(√) any two** that motivated you to choose a preferred specialty field of study

| **No** | **Organization related factors** | **Indicate choices by(√)** |
| --- | --- | --- |
| **1** | Opportunities for higher qualifications |  |
| **2** | Specialty training/ curriculum |  |
| **3** | Good professional exposure |  |
| **4** | Good pay in a field/lucrative salary |  |
| **5** | Length of postgraduate training |  |
| **6** | None of the above is applicable |  |

**C 2.2** For the **Socio-cultural factors** listed below; please mark **(√) at least any two** that motivated you to choose a preferred specialty field of study

| **No** | **Socio-cultural factors** | **Indicate choices by(√)** |
| --- | --- | --- |
| **1** | Respect |  |
| **2** | Family needs |  |
| **3** | Social rewards |  |
| **4** | Religious believes |  |
| **5** | Cultural believes |  |
| **6** | None of the above is applicable |  |

**C 2.3** For **the Intrinsic factors** listed below, please mark **(√) any two** that motivated you to choose a preferred specialty field of study

| **No** | **Intrinsic Factors** | **Indicate choices by(√)** |
| --- | --- | --- |
| **1** | Empowerment |  |
| **2** | Autonomy/Individual goal |  |
| **3** | Career growth |  |
| **4** | Training opportunities |  |
| **5** | Work interest/ Serving people |  |
| **6** | None of the above is applicable |  |

**Section D: Job Preferences:**

**D1.** Which sector do you prefer to work after internship? 1. Public ☐ 2. Private ☐

**D2.** Which of the factors listed below will influence your choice for sector of practice? Please tick **(√)** ‘Yes’ or ‘No’ (select one choice for each statement)

| **Factor affecting choice for sector of practice** | **Yes** | **No** |
| --- | --- | --- |
| 1. Opportunities for professional development |  |  |
| 2. Good working condition, including better infrastructure and equipment and good hygienic condition |  |  |
| 3. Workplace security |  |  |
| 4. Salaries and financial incentives |  |  |
| 5. Social Respect |  |  |
| 6. Family Influence |  |  |
| 7. Willingness to work for the marginalized population |  |  |
| 8. Autonomy and Self empowerment |  |  |
| 9. Ability to support oneself and the family |  |  |
| 10. Job Security |  |  |

**Section E: Location of Practice:**

**E1.** Where do you intend to practice after internship? 1. Rural ☐ 2. Urban/Small town ☐
**E2.** Which of the factors listed below will influence your choice for location of practice? Please tick **(√)** ‘Yes’ or ‘No’ (select one choice for each statement

| **Factor affecting choice of location** | **Yes** | **No** |
| --- | --- | --- |
| 1. Access to social and family networks |  |  |
| 1. Adventure & recreational opportunities |  |  |
| 1. Access to Information and Communication Technology |  |  |
| 1. Road Infrastructure |  |  |
| 1. Community belonging |  |  |
| 1. Good working condition, including better infrastructure and equipment and good hygienic condition |  |  |
| 1. Workplace security |  |  |
| 1. Opportunities for children |  |  |
| 1. Opportunities for own continuing education |  |  |
| 1. Opportunities for partner/spouse |  |  |
| 1. Rural lifestyle |  |  |

**E3.** Where do you intend to obtain further training? 1. Bangladesh ☐ 2. Abroad ☐

**E4.** Where do you intend to spend majority of your working life after specialist training?

1. Bangladesh ☐ 2. Abroad ☐

**If the answer of E4 is ‘Bangladesh’, then skip the next question (E5) and go to E6.**

**E5.** In abroad, where do you intend to work?
1. USA ☐ 2. UK ☐ 3. Canada ☐ 4. European Union Countries ☐ 5. Australia ☐ 6. Singapore ☐ 7. Middle East ☐ 8. Any countries which pays better ☐ 9. Where husband/ family resides ☐ 10. Not applicable ☐

**E6.** Which of the factors listed below will influence your choice for country of practice? Please tick **(√)** ‘Yes’ or ‘No’ (select one choice for each statement)

| **Factor affecting choice for country of practice** | **Yes** | **No** |
| --- | --- | --- |
| 1. Access to social and family networks |  |  |
| 1. Adventure & recreational opportunities |  |  |
| 1. Availability of other specialists |  |  |
| 1. Community belonging |  |  |
| 1. Opportunities for children |  |  |
| 1. Opportunities for own continuing education |  |  |
| 1. Opportunities for partner/spouse |  |  |
| 1. Political stability |  |  |
| 1. Quality of work facilities |  |  |
| 1. Registration and Accreditation procedure |  |  |
| 1. Salary |  |  |
| 1. Safety and security issues |  |  |

**Thank You for Your Participation**
