# Supplementary material for: ‘Feminization’ of physician workforce in Bangladesh, underlying factors and implications for health system: Insights from a mixed-methods study
Source: PLoS One. 2019 Jan 11;14(1):e0210820. doi: 10.1371/journal.pone.0210820 (PMC6329528; doi:10.1371/journal.pone.0210820)
Supplement: S2 File — (DOCX) [file pone.0210820.s002.docx]

**Qualitative Guidelines**

1. **IDI Guideline for Female Medical Students in English**

| **Guidelines for In depth-Interview Female Students** | | | |
| --- | --- | --- | --- |
| Date/Duration: ……………………… | | | |
| Start time: ……………. | Time finished: ………… | | Total duration: ……. |
| Facilitator: ………………………… | | Note Taker: ………………………… | |
| **What are the familial factors that influenced you to pursue medical education?**  Probes:   - Did you come by your own wish? - When did you start to think of being a doctor? - What was your thinking about medical education? - Are there any other familial factors that influenced you? - What was your parent’s opinion about medical education? - Who influenced you most? - How did they influence you, can you please explain? - What is your opinion about your family influence?   **What are the factors that motivated you individually?**  Probes:   - Do you have any personal motivation for studying medicine? - What are those factors? - Why those factors are important to you? - What was your preference in admission (public/ private)? - Why did you prefer this medical for admission (public/ private) (i.e.: Distance, living quality inside/ outside Dhaka, hostel facility, living with family, academic environment between public and private)?   **What are social factors for choosing medical education?**  Probes:   - Are there any reasons beside the individual and familial factors? - What about the society around you? (i.e.: Neighborhood pressure, competition with others, desire to stand in a better position, maintaining family status in the society) - Is there any social factor that influenced you? - Did you have any motive for the society? (Service to the society, helping poor, and working for the native villagers)? | | | |
| **What was your perception about medical school and how do you feel about your student life?** | | | |
| ***Probes:*** | | | |
| - Why did you come to medical school? - What was your expectation before coming to medical school? - How do you feel about your student life? - Did you face any obstacles during your study? (i.e. academic, institutional, social and familial) | | | |
| **Would you please share with us your future plan?** | | | |
| ***Probes:*** | | | |
| - What are your plans after completion of studies? (i.e. urban/rural, public/private, migration) - How do you want to proceed with your future plans? | | | |
| **What do you think about the current ratio of male to female in medical schools?** | | | |
| ***Probes:*** | | | |
| - What is the male to female ratio in your class? - What is your perception about the number of females? - Why do you think females are coming into medical profession? - How do you feel about female doctors pursuing their career? | | | |
| **What is your perception about the challenges of medical schools and studies?** | | | |
| ***Probes:*** | | | |
| - What are the challenges of medical students? - Do you feel that the challenges are different for females? - How do you cope with the challenges? | | | |
| **What is your perception about the drop out from medical profession?** | | | |
| ***Probes:*** | | | |
| - Do you know about drop out from medical studies and profession? Why do you think that people drop out from medical studies and profession? - What is your perception about the trend of drop out? - Why do female students and doctor drop out? - Do you think it will have any effect on the health sector? | | | |
|  | | | |
| **Would you please share with us the plan that where you want to work (location: urban/ rural) after completion of your final course?** | | | |
| ***Probes:*** | | | |
| - Why you want to work in urban area/ rural area? - What are the factors which motivated you to take this decision? - Would you please share your opinion about rural retention of female medical doctors? - What are the challenges a female doctor faces during rural deployment? | | | |
| **Would you please share with us in which sector you want to work after completion of MBBS? (public/ private)** | | | |
| ***Probes:*** | | | |
| - Why you want to work in public/private service? - What are the factors which motivated you to take this decision? - Would you please share your opinion about working in public sector as a female medical doctor? - What is your opinion about working in private sector as a female medical doctor? | | | |
| **Would you please share with us plan regarding migration to another country?** | | | |
| ***Probes:*** | | | |
| - If you want to migrate, why? - What are the real intentions for migration?   If Permanent------Why ?  If Temporary-------why ?   - Any possibility of return back or to settle there? | | | |

1. **IDI Guideline for Female Medical Students in Bangla**

| **নিবিড় সাক্ষাৎকার – ছাত্রী** |
| --- |
| **তারিখঃ** |
| **সাক্ষাৎকার শুরুর সময়ঃ সাক্ষাৎকার শেষের সময়ঃ** |
| **সাক্ষাৎকার গ্রহণকারীঃ তথ্য গ্রহণকারীঃ** |
| **কি কি পারিবারিক ব্যাপার আপনাকে মেডিকেল এ পড়ালেখার জন্য প্রভাবিত করেছিল?**  **প্রোবঃ**   - আপনি কি নিজের ইচ্ছায় মেডিকেল এ এশেছিলেন? - আপনি কখন চিকিৎসক হওয়ার জন্য চিন্তাভাবনা শুরু করেন? - মেডিকেল শিক্ষার ব্যাপারে আপনার অভিমত কি ছিল? - কোন **পারিবারিক বিষয় কি আপনাকে মেডিকেল কলেজে পড়তে উদ্ভুদ্ধ করেছে?** - মেডিকেল এ পড়ালেখার ব্যাপারে আপনার বাবা মায়ের মতামত কি ছিল? - কে আপনাকে বেশি প্রাবিতভ করেছিল? - ওনারা/ উনি কিভাবে আপনাকে প্রভাবিত করেছিল? - মেডিকেলে পড়ার বিষয়ে পারিবারিক প্রভাব সম্পর্কে আপনার মতামত কি?   **ব্যক্তিগত ভাবে কোন কোন বিষয় আপনাকে মেডিকেল এ ভর্তির জন্য অনুপ্রাণিত করেছিল?**  **প্রোবঃ**   - মেডিকেল এ পড়ার জন্য কোন বাক্তিগত বিষয় কি আপনাকে অনুপ্রাণিত করেছিল? - এই বিষয় গুলো কি কি? - এই বিষয় গুলো আপনার কাছে কেন গুরুত্বপূর্ণ মনে হয়েছে? - আপনি কোথায় ভর্তি হতে চেয়েছিলেন? (সরকারী না বেসরকারী মেডিকেল কলেজ)? - আপনার পরিবারের ইচ্ছা ছিল কোথায় ভর্তি হওয়া (সরকারী না বেসরকারী মেডিকেল কলেজ)? - কেন? (ঢাকা/ ঢাকার বাইরে জীবনযাত্রার মান, হোস্টেলে সুবিধা, পরিবারের সাথে থাকা, পড়াশুনার পরিবেশ, দূরত্ব)   সামাজিক কি কি কারণ আপনাকে মেডিকেল কলেজে পড়তে অনুপ্রাণিত করেছে?  **প্রোবঃ**   - পারিবারিক বা **ব্যক্তিগত কারণের বাইরে আর কোন কারণ রয়েছে?** - আপনার পারিপার্শ্বিক সমাজ ব্যবস্থা এই বিষয়ে কোন প্রভাব বিস্তার করেছে (যেমনঃ আশেপাশের চাপ, অন্যের সাথে প্রতিযোগিতা, ভালো একটা অবস্থানে যাওয়া, পারিবারিক মর্যাদা)? - সমাজের প্রতি আপনার কোন কর্তব্যবোধ থেকে কোন সিদ্ধান্ত নিয়েছেন (যেমনঃ সমাজের প্রতি দায়িত্ববোধ, গরীব মানুষকে সহায়তা করা, নিজের গ্রামের মানুষ এবং সমাজকে সহায়তা করা)? |
| **মেডিকেল কলেজ সম্পর্কে আপনার কি ধারণা ছিল এবং আপনি আপনার ছাত্রজীবন সম্পর্কে কি অনুভব করেন?** |
| **প্রোবঃ** |
| - আপনি কেন চিকিৎসাবিদ্যায় আগ্রহী হলেন? - আপনার প্রত্যাশা কি ছিল? - ছাত্র জীবন সম্পর্কে আপনার মতামত কি? - আপনি কি কোন ধরনের প্রতিকূলতার সম্মুখীন হয়েছেন? (একাডেমিক, প্রাতিষ্ঠানিক, সামাজিক, পারিবারিক) |
| **আপনার ভবিষ্যৎ পরিকল্পনা কি?** |
| **প্রোবঃ** |
| - পড়াশোনা শেষে আপনার পরিকল্পনা কি? - আপনি কিভাবে সে বিষয়ে অগ্রসর হতে চান? |
| **বর্তমানে চিকিৎসাশাস্ত্রে নারী ও পুরুষদের অংশগ্রহণের অনুপাত সম্পর্কে আপনার কি মতামত?** |
| **প্রোবঃ** |
| - আপনার ক্লাসে নারী ও পুরুষের অনুপাত কি? - নারীদের অংশগ্রহণের অনুপাত সম্পর্কে আপনার ধারণা কি? - নারীদের চিকিৎসাশাস্ত্রে আসার কারণ কি বলে মনে করেন? - নারীদের চিকিৎসা কে পেশা হিসেবে নেয়ার ব্যাপারে আপনার মতামত কি? |
| **চিকিৎসা শিক্ষায় কি ধরনের প্রতিকূলতা আছে বলে আপনার ধারণা?** |
| **প্রোবঃ** |
| - চিকিৎসা শাস্ত্রের শিক্ষার্থীদের কি ধরনের প্রতিকূলতার সম্মুখীন হতে হয়? - নারীদের জন্য কি প্রতিকূলতা ভিন্ন? - কিভাবে এই প্রতিকূলতা মানিয়ে নেয়া/ অতিক্রম করা সম্ভব? |
| **চিকিৎসা শাস্ত্র বিদ্যা থেকে ঝরে পড়া সম্পর্কে আপনার কি মতামত?** |
| **প্রোবঃ** |
| - আপনি কি চিকিৎসাশাস্ত্র থেকে ঝরে পড়ার ব্যাপারে অবহিত? - আপনার কি ধারণা কেন ছাত্ররা চিকিৎসাশাস্ত্র বিদ্যা থেকে ঝরে পড়ে? - ঝরে পড়ার ধারা সম্পর্কে আপনি কি জানেন? এই বিষয়ে আপ্নার মতামত কি? - কেন ছাত্রী ও নারী চিকিৎসকরা ঝরে পড়েন? - আপনার কি মনে হয় স্বাস্থ্য খাতে এর কোন প্রভাব পড়বে? |
| \| এমবিবিএস শেষ করার পর আপনি কোন এলাকায় (শহর / গ্রাম) কাজ করতে চাচ্ছেন? \| \| --- \| \| **প্রোবঃ** \| \| - আপনি কেন শহর/ গ্রামাঞ্চলে কাজ করতে চান? - এই সিদ্ধান্ত নিতে কি কি বিষয় আপনাকে অনুপ্রাণিত করেছেন? - নারী চিকিৎসক হিসেবে গ্রামাঞ্চলে কাজ করা নিয়ে করা নিয়ে আপনার মতামত বলুন। - একজন নারী চিকিৎসক গ্রামাঞ্চলে কাজ করতে গিয়ে কি কি অসুবিধার সম্মুখীন হন? \| \| এমবিবিএস শেষ করার পর আপনি কোন সেক্টরে কাজ করতে চাচ্ছেন (সরকারী/ বেসরকারী)? \| \| **প্রোবঃ** \| \| - আপনি কেন সরকারী/ বেসরকারী সেক্টরে কাজ করতে চান? - এই সিদ্ধান্ত নিতে কি কি বিষয় আপনাকে অনুপ্রাণিত করেছেন? - নারী চিকিৎসক হিসেবে সরকারী সেক্টরে কাজ করা নিয়ে করা নিয়ে আপনার মতামত বলুন। - নারী চিকিৎসক হিসেবে বেসরকারী সেক্টরে কাজ করা নিয়ে করা নিয়ে আপনার মতামত বলুন। \| \| **অন্য কোন দেশে অভিবাসন/ মাইগ্রেট করার বিষয়ে আপনার কোন পরিকল্পনা রয়েছে?** \| \| **প্রোবঃ** \| \| - যদি আপনার পরিকল্পনা থাকে, আপনি কোথায় অভিবাসন/ মাইগ্রেট করতে চাচ্ছেন, কেন? - আপনার অভিবাসনের আসল উদ্দেশ্য কি?   যদি স্থায়ী হয়, কেন?  যদি অস্থায়ী হয়, কেন?   - দেশের বাইরে গেলে কি ফিরে আসবেন নাকি সেখানেই থেকে যাবেন? \| |

1. **FGD Guideline for Female Medical Students in English**

| **Guidelines for Focus Group Discussion Female students** | | | |
| --- | --- | --- | --- |
| Date/Duration: ……………………… | | | |
| Start time: ……………. | Time finished: ………… | | Total duration: ……. |
| Facilitator: ………………………… | | Note Taker: ………………………… | |
| **What is the situation of female medical students of Bangladesh?** | | | |
| ***Probes:*** | | | |
| - Why did you come to medical education? - What was the expectation? - What were the realities of student life? - How do you feel about women coming into medicine? - Do you think more women are coming into medicine than before? - Why do you think females are coming more? | | | |
| **What is the situation of female student who is going towards professional life after completion of studies?** | | | |
| ***Probes:*** | | | |
| - What was the expectation from medical studies? - Does reality match with the perception? - What are the challenges that female doctors face while looking for employment? - After employment, did female doctors face different challenges from male doctors? | | | |
| **What are the challenges that the female doctors face?** | | | |
| ***Probes:*** | | | |
| - What are the challenges of medical education? - What are the challenges during career for a medical professional? - What is you perception about the different challenges that a female faces during studies and career? Does it differ from a male? How? - How do a female medical student and a female doctor cope up with the challenges? | | | |
| **What is your opinion about female doctors not pursuing their studies and career?** | | | |
| ***Probes:*** | | | |
| - Do you know about the drop out of fellow female students and doctors? - Why do they drop out? - What are the other factors associated with drop out? - Do you think it will have any effect on the current scenario? - How can it be reduced? | | | |
| **What are the factors influencing feminization of medicine among female medical student?** | | | |
| ***Probes:*** | | | |
| - How is medical study so far? - Are you enjoying it, much as you expected? - How many of you in class? - What’s the student’s distribution in terms of male and female? - As a group in your own view why is more women are choosing medicine as a career? - Would you share your opinion about feminization of medicine and the associated factors influencing career choice in medicine and future specialty? - Can you through light on some of the reasons/factors you just mentioned? - Is the learning environment more accommodating than before? - Do you think the feminization of medicine occurrence has anything to do with the learning environments? (probe) - Finally, as a group what do you think will be the future of medicine in this country? | | | |

1. **FGD Guideline for Female Medical Students in Bangla**

| **ফোকাস গ্রুপ ডিস্কাশন গাইডলাইন – ছাত্রী** |
| --- |
| তারিখঃ |
| সাক্ষাৎকার শুরুর সময়ঃ সাক্ষাৎকার শেষের সময়ঃ |
| সাক্ষাৎকার গ্রহণকারীঃ তথ্য গ্রহণকারীঃ |
| **বাংলাদেশে চিকিৎসা শাস্ত্রে নারী শিক্ষার্থীদের সার্বিক পরিস্থিতি কি?** |
| **প্রোবঃ** |
| - কেন চিকিৎসাশাস্ত্রে আগ্রহী হলেন?(এইচ এস সি র পরে) - কি ধরনের প্রত্যাশা ছিল? - চিকিৎসাশাস্ত্রের বাস্তবতা কেমন? - চিকিৎসাশাস্ত্রে নারী শিক্ষার্থীদের আগ্রহ সম্পর্কে আপনার কি মতামত? - চিকিৎসা শাস্ত্রে বর্তমানে নারী শিক্ষার্থীদের অংশগ্রহণ কেমন? - আপনার কি ধারণা, নারীরা অতীতের থেকে বেশি আগ্রহী? - আপনার কি ধারণা, কেন নারীরা চিকিৎসা শাস্ত্রে নারীরা বেশি আগ্রহী হচ্ছে? |
| **একজন নারী শিক্ষার্থী যে শীঘ্রই চাকরি জীবনে প্রবেশ করবে তার পড়াশোনা শেষে পরিস্থিতি কি?** |
| **প্রোবঃ** |
| - মেডিকেলে ভর্তি হবার পর কি ধরনের প্রত্যাশা ছিল? - বাস্তবতা কেমন? - একজন নারীকে চাকরি খোঁজার ক্ষেত্রে কি কি ধরনের প্রতিকূলতার সম্মুখীণ হতে হয়? - চাকরি পাওয়ার পরে কি কি ধরনের প্রতিকূলতার মুখোমুখি হতে হয়? তা কি পুরুষ সহকর্মী থেকে ভিন্ন? |
| **একজন নারী চিকিৎসক কে কি কি ধরনের প্রতিকূলতার মুখে পড়তে হয়?** |
| **প্রোবঃ** |
| - চিকিৎসা শাস্ত্র শিক্ষার ক্ষেত্রে কি কি ধরনের প্রতিকূলতা আছে? - চিকিৎসা পেশায় কি কি ধরনের প্রতিকূলতা আছে? - একজন নারী শিক্ষার্থী তার শিক্ষা জীবনের কি কি ধরনের প্রতিকূলতার মুখোমুখি হয়? তা কি একজন পুরুষের থেকে ভিন্ন? হলে কিভাবে? - একজন নারী শিক্ষার্থী কিভাবে এই প্রতিকূলতা অতিক্রম করেন? - একজন নারী চিকিৎসক কিভাবে এই প্রতিকূলতা অতিক্রম করেন? |
| **একজন নারী কেন চিকিৎসাশাস্ত্র বিদ্যা ও চিকিৎসা পেশা থেকে ঝরে পড়েন?** |
| **প্রোবঃ** |
| - আপনি কি নারী শিক্ষার্থী ও চিকিৎসক দের ঝরে পড়া সম্পর্কে অবহিত? - কেন তারা ঝরে পড়েন? - আর কি কি বিষয় এই ঝরে পড়ার সাথে জড়িত? - আপনার কি মনে হয়, এই ঝরে পড়া ভবিষ্যতে সাস্থ্য খাতে কোন প্রভাব ফেলবে? - কিভাবে এটা প্রশমিত করা সম্ভব? |
| চিকিৎসাশিক্ষায় নারীর ক্রমবর্ধমান উপস্থিতির কারণ কি কি? |
| **প্রোবঃ** |
| - এত দিন চিকিৎসা শিক্ষা আপনাদের কাছে কেমন লেগেছে? - আপনি কি এটা উপভোগ করেছেন? এটা কি আপনার প্রত্যাশা অনুযায়ী ছিল? - আপনাদের ক্লাশে শিক্ষার্থী কতজন? - শিক্ষার্থীদের মাঝে নারী পুরুষের অনুপাত কেমন? - গ্রুপ হিসেবে আপনাদের কি মনে হয়, কেন নারিরা বেশি চিকিৎসাপেশায় আসছেন? - চিকিৎসাপেশায় নারীর ক্রমবর্ধমান উপস্থিতি এবং এর সাথে বিষয়/ স্পেসালিটি নির্বাচনের উপর কি কি প্রভাব ফেলে? আপনার মতামত বলুন। - এই সম্পর্কে কিছু কারণ/ প্রভাবক বলতে পারবেন? - শেখার পরিবেশ কি আগের তুলনায় ভালো? - শেখার পরিবেশের সাথে চিকিৎসাপেশায় নারীর ক্রমবর্ধমান উপস্থিতি কোন সম্পর্ক রয়েছে? - গ্রুপ হিসেবে আপনাদের কি মনে হয়, এই দেশে চিকিৎসাপেশার ভবিষ্যৎ কি? |

1. **IDI Guideline for Female In-service Trainees in English**

| **Guidelines for Female In-service Trainee Physicians** | | | |
| --- | --- | --- | --- |
| Date/Duration: ……………………… | | | |
| Start time: ……………. | Time finished: ………… | | Total duration: ……. |
| Facilitator: ………………………… | | Note Taker: ………………………… | |
| **What was your perception about medical school and how do you feel about your student life?** | | | |
| ***Probes:*** | | | |
| - Why did you come to medical school? - What was your expectation? - Has your expectation been fulfilled? If yes, why, if not, why? - Did you face any obstacle during student life? | | | |
| **What was your experience once you finished studies?** | | | |
| ***Probes:*** | | | |
| - Did you face any obstacle during your internship? If yes, then what type of difficulties? - How did you cope up with the challenge/ obstacle? | | | |
| **What do you feel about the current trend of female admission in medical studies?** | | | |
| ***Probes:*** | | | |
| - Do you think things have changed from the time you got admitted? - What do you think about women coming to medicine? - In your opinion what is the reason behind females choosing medical profession? | | | |
| **What do you think about the drop out of females from medical profession?** | | | |
| ***Probes:*** | | | |
| - Do you know about drop out of female doctors? - What are the factors associated with it? - How can it be reduced? | | | |

1. **IDI Guideline for Female in service Trainnes in Bangla**

| **নারী শিক্ষানবিশ ডাক্তারের নিবিড় সাক্ষাৎকার গাইডলাইন** |
| --- |
| তারিখঃ |
| সাক্ষাৎকার শুরুর সময়ঃ সাক্ষাৎকার শেষের সময়ঃ |
| সাক্ষাৎকার গ্রহণকারীঃ তথ্য গ্রহণকারীঃ |
| **মেডিকেল কলেজ সম্পর্কে আপনার কি ধারণা ছিল এবং আপনি আপনার ছাত্রজীবন সম্পর্কে কি অনুভব করেন?** |
| **প্রোবঃ** |
| - আপনি কেন চিকিৎসাবিদ্যায় আগ্রহী হলেন? - আপনার প্রত্যাশা কি ছিল**?** - আপনার প্রত্যাশা কি পূরণ হয়েছে? যদি হ্যাঁ, তবে কেন? যদি না, তবে কেন? - আপনি কি ছাত্রজীবনে কোন প্রতিকূলতার সম্মুখীন হয়েছেন? |
| **ছাত্রজীবন শেষে আপনার অভিজ্ঞতা কেমন ছিল?** |
| **প্রোবঃ** |
| - আপনি কি ইন্টার্নশিপের সময় কোন প্রতিকূলতার মুখোমুখি হয়েছেন? যদি হ্যাঁ, তবে কিধরনের? - আপনি কিভাবে এই প্রতিকূলতা অতিক্রম করলেন? |
| **বর্তমানে চিকিৎসাশাস্ত্রে নারীদের অংশগ্রহণ সম্পর্কে আপনার কি মতামত?** |
| **প্রোবঃ** |
| - আপনার ভর্তির সময় থেকে কি পরিস্থিতির পরিবর্তন হয়েছে? - নারীদের চিকিৎসা পেশায় আগমন সম্পর্কে আপনার কি মতামত? - এর পেছনে কি কারণ কাজ করছে বলে আপনি মনে করেন? |
| **নারীদের চিকিৎসাপেশা থেকে ঝরে পড়া সম্পর্কে আপনার কি মতামত?** |
| **প্রোবঃ** |
| - নারীদের ঝরে পড়া সম্পর্কে কি আপনি অবহিত? - এর পেছনে কি কারণ কাজ করছে বলে আপনি মনে করেন? - কিভাবে এর প্রশমন সম্ভব? |

1. **IDI Guideline for Parents of female medical students in English**

| **Guidelines for Parents of female medical students** | | | |
| --- | --- | --- | --- |
| Date/Duration: ……………………… | | | |
| Start time: ……………. | Time finished: ………… | | Total duration: ……. |
| Facilitator: ………………………… | | Note Taker: ………………………… | |
|  | |  | |

- **Explore general information about the respondent**
- Relation with the student/doctor, Number of Family members, Earning per week/month
- Explore about composition of family members; bread winners of the family
- **How do you feel being a parent of a female doctor?**
- As a parent do you find your daughter’s journey difficult?
- Has it changed or is going to change your life anyway?
- **What made you to enrol your daughter to this medical profession?**
- Why did you find this profession suitable for your daughter?
- Was there any other point that made you to select this?
- Do you think that any gender should be enrolled more in this profession?
- **Have being a medical student/doctor made any change in your daughter’s life?**
- What’s about their family life?
- Can this profession affect in economic ground or bride price anyway?
- **If you are a patient then which gender doctor you prefer to visit?**
- Do you find any difference in between male and female doctors?
- If yes, why?
- **What do you think that female doctors should think while choosing their career or speciality?**
- What do you think about their family life and career choosing?
- Where do you want to see your daughter?

1. **IDI Guideline for Parents of female medical students in Bangla**

| **নারী মেডিকেল শিক্ষার্থীর বাবা মা/ অভিভাবকের নিবিড় সাক্ষাৎকার গাইডলাইন** |
| --- |
| তারিখঃ |
| সাক্ষাৎকার শুরুর সময়ঃ সাক্ষাৎকার শেষের সময়ঃ |
| সাক্ষাৎকার গ্রহণকারীঃ তথ্য গ্রহণকারীঃ |
| **উত্তর দাতার সাধারণ তথ্য** |
| **প্রোবঃ** |
| - বয়স, ধর্ম, পরিবারের সদস্য সংখ্যা , সাপ্তাহিক/ মাসিক আয়। - পরিবারের সদস্যদের বিষয়ে আরও জানা, পরিবারের প্রধান আয়কারীর তথ্য |
| একজন নারী চিকিৎসক এর অভিভাবক হিসাবে আপনার অনুভুতি কেমন? |
| **প্রোবঃ** |
| - আপনার কাছে কি তার এই মেডিকেল এর শিক্ষাজীবন কঠিন বলে মনে হয়েছে? - একজন নারী চিকিৎসক এর অভিভাবক হিসেবে এটি কি আপনার কোনো প্রভাব বা পরিবর্তন এনেছে? |
| আপনি কি কারনে আপনার মেয়েকে মেডিকেল শিক্ষায় ভর্তি করালেন? |
| **প্রোবঃ** |
| - আপনার কাছে আপনার মেয়ের জন্য এই পেশা উপযুক্ত কেন মনে হল? - আর কোন কারন কি ছিল এই পেশা পছন্দ করার জন্য? - আপনি কি মনে করেন কোনো নির্দিষ্ট লিঙ্গের মানুষের এই পেশায় বেশী আসা উচিত? |
| মেডিকেল স্টুডেন্ট/ডাক্তার হওয়াতে আপনার মেয়ের জীবনে কি কোন পরিবর্তন এসেছে? |
| **প্রোবঃ** |
| - তাদের পারিবারিক জীবনে কি কোনো পরিবর্তন এসেছে? - এই পেশা কি কোন ভাবে তাদের জীবনে অর্থনৈতিক ভাবে কোনো পরিবর্তন এসেছে?বা পাত্রী হিসেবে? |
| আপনি একজন রোগী হলে কোন লিঙ্গের ডাক্তার এর কাছে যেতে পছন্দ করতেন? |
| **প্রোবঃ** |
| - আপনি কি পুরুষ ও মহিলা ডাক্তার এর মধ্যে কোন পার্থক্য পান? - যদি হ্যাঁ হয়, তবে কেন? |
| আপনি কি ভাবেন, মহিলা ডাক্তার দের তাদের ভবিষ্যৎ ক্যারিয়ার ও বিষয় পছন্দ করার সময় কি কি বিষয় বিবেচনা করা উচিত? |
| **প্রোবঃ** |
| - আপনি তাদের পারিবারিক জীবন ও ক্যারিয়ার পছন্দ করার বেপারে কি ভাবেন? - আপনি আপনার মেয়েকে ভবিষ্যতে কোথায় দেখতে চান? |
